# Non-HLA Antibodies in Kidney Transplantation: Mediators of Allograft Injury or Markers of Dysregulated Humoral Immunity?

**DOI:** 10.3390/medsci14050534

**Published:** 2026-08-31

**Authors:** Ariadni Fouza, Maria Daoudaki

**Affiliations:** 1 5th Surgical Department, Hippokratio General Hospital, School of Medicine, Aristotle University of Thessaloniki, 54642 Thessaloniki, Greece; ariadnefou@gmail.com; 2 Department of Transplant Surgery, Center for Research and Innovation in Solid Organ Transplantation, School of Medicine, Aristotle University of Thessaloniki, 54124 Thessaloniki, Greece; 3 Laboratory of Biological Chemistry, School of Medicine, Aristotle University of Thessaloniki, University Campus, 54124 Thessaloniki, Greece

**Keywords:** non-HLA antibodies, kidney transplantation, antibody-mediated rejection, humoral immunity, endothelial injury, precision immune monitoring

## Abstract

Antibody-mediated rejection (AMR) remains a major cause of kidney allograft injury and loss. Although donor-specific antibodies against human leukocyte antigens (HLA-DSAs) are central to humoral alloimmunity, they do not explain all cases of microvascular inflammation or graft dysfunction. This review critically evaluates non-HLA antibodies as potential mediators of allograft injury and markers of dysregulated humoral immunity by integrating functional, experimental, clinicopathological, and clinical evidence. Receptor-targeting antibodies, especially those against the angiotensin II type 1 receptor (AT1R) and the endothelin-1 type A receptor (ETAR), have the strongest evidence for direct pathogenicity. Antibodies against major histocompatibility complex class I-related chain A (MICA) and glutathione S-transferase theta-1 (GSTT1) reflect non-HLA alloimmunity, whereas antibodies targeting perlecan-derived LG3, vimentin, and injury-associated antigens may arise through secondary autoimmunity and mark broader humoral activation. We propose that selected pathogenic non-HLA antibodies participate in a feed-forward cycle of graft injury, antigen exposure, epitope spreading, and humoral amplification, although this model requires longitudinal validation. Crucially, non-HLA antibody positivity alone should neither establish AMR nor guide antibody-specific treatment. Its interpretation should be target-specific and integrated with HLA-DSAs, histopathology, molecular findings, graft function, and evidence of immune activation.

## 1. Introduction

Antibody-mediated rejection (AMR) remains a major barrier to long-term kidney allograft survival despite advances in immunological risk assessment, donor–recipient matching, and immunosuppressive therapy [1]. The humoral alloimmune response has traditionally been understood primarily through donor-specific antibodies against human leukocyte antigens (HLA-DSAs), which are strongly associated with microvascular inflammation, complement activation, transplant glomerulopathy, and progressive graft loss [1,2]. However, HLA-DSAs alone do not fully explain the complexity of antibody-mediated allograft injury. Some kidney transplant recipients develop histological features consistent with AMR in the absence of detectable circulating HLA-DSAs, whereas others maintain persistent HLA-DSAs for years without clinically significant graft dysfunction. These observations suggest that additional humoral mechanisms contribute to allograft injury and challenge the traditional HLA-centric paradigm of transplant immunology [2,3].

This broader understanding is reflected in the Banff classification, which allows circulating donor-specific antibodies against HLA or other antigens to support the diagnosis of AMR when interpreted within the appropriate clinicopathological context [4,5]. Nevertheless, the clinical interpretation of non-HLA antibodies remains challenging because of limited assay standardization, inconsistent thresholds, and considerable heterogeneity in the evidence supporting individual antibody specificities. Unlike HLA antibodies, non-HLA responses may arise through the recognition of genetically disparate donor antigens or through injury-associated exposure or modification of self-antigens. They therefore lie at the intersection of alloimmunity, autoimmunity, and tissue injury [6]. Relevant targets include genetically disparate donor antigens, such as major histocompatibility complex class I-related chain A (MICA) and mismatch-associated glutathione S-transferase theta-1 (GSTT1), as well as self-antigens exposed, released, or modified during graft injury [3,7].

The biological and clinical significance of non-HLA antibody positivity is increasingly recognized but remains target- and context-dependent. Selected non-HLA antibodies appear capable of directly inducing endothelial activation, inflammatory signaling, and vascular injury, whereas others may primarily reflect ongoing tissue damage or broader alterations in humoral immunity. Non-HLA antibodies may therefore function as pathogenic effectors, indicators of altered B-cell homeostasis, or both [6,7,8]. This distinction has important biological and clinical implications. If selected antibodies directly mediate allograft injury, they may represent therapeutic targets; if they predominantly reflect an altered humoral immune state, their principal value may lie in immunological risk stratification. Distinguishing between these possibilities is central to the appropriate interpretation of non-HLA antibody testing.

We propose that broad non-HLA antibody repertoires may reflect underlying humoral immune dysregulation, whereas selected pathogenic antibodies may also contribute to its persistence by promoting further graft injury and antigen exposure. Dysregulated humoral immunity is therefore used here as a working model integrating persistent antigenic stimulation, loss of self-tolerance, antibody-repertoire diversification, and sustained humoral activation. Nevertheless, the proposed relationship between broad non-HLA antibody repertoires and defined B-cell states remains hypothesis-generating and requires longitudinal validation.

**Figure 1:**
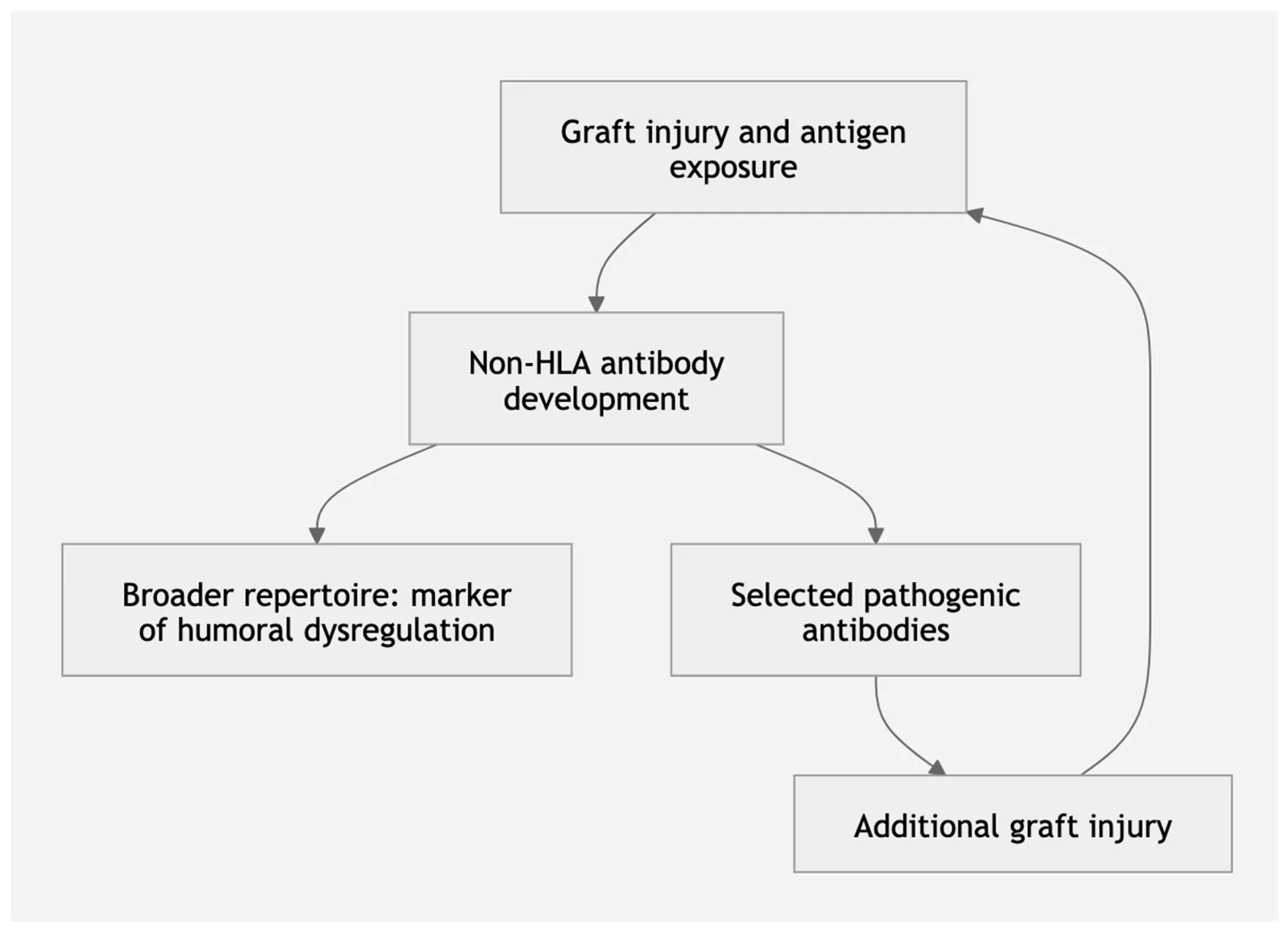
Proposed dual interpretation of non-HLA antibody responses. Broader repertoires may reflect humoral immune dysregulation, whereas selected pathogenic antibodies may amplify graft injury and antigen exposure through a potential feed-forward cycle.

The purpose of this review is to provide a critical and integrated assessment of the biological and clinical significance of non-HLA antibodies in kidney transplantation. We evaluate the evidence supporting individual antibody specificities as potential mediators of allograft injury or markers of humoral immune dysregulation, examine the pathways underlying their development and their interactions with HLA-DSAs and consider their potential contribution to precision immune monitoring. Particular attention is given to the distinction between selected antibodies with plausible pathogenic activity and broader antibody repertoires that may reflect sustained humoral immune activation. We also propose a potential feed-forward relationship linking antibody-mediated injury, secondary antigen exposure, epitope spreading, and humoral amplification, as summarized in Figure 1.

### Literature Search Strategy

A structured literature search was conducted in PubMed, Scopus, and Web of Science for articles published up to June 2026. Searches combined Medical Subject Headings and free-text terms, including “non-HLA antibodies,” “kidney transplantation,” “antibody-mediated rejection,” “AT1R,” “MICA,” “vimentin,” and “humoral dysregulation.” Priority was given to peer-reviewed clinical studies, multicentre observational cohorts, validation studies, and in vivo experimental models providing relevant mechanistic or clinical evidence. Studies were selected according to their relevance to kidney transplantation, methodological quality, and contribution to understanding antibody detection, pathogenic mechanisms, clinicopathological associations, or graft outcomes. Evidence from other transplant settings was included only when it provided directly applicable biological evidence.

## 2. Immunological Mechanisms Underlying Non-HLA Humoral Immunity

The development of non-HLA antibodies cannot be attributed to a single immunological pathway. Unlike donor-specific HLA antibodies, which arise predominantly through classical alloimmune recognition of donor HLA molecules, non-HLA humoral immunity develops within a dynamic graft environment shaped by ischemia–reperfusion injury, innate and adaptive immune activation, endothelial remodeling, chronic inflammation, and progressive alterations in B-cell homeostasis [3,9]. Rather than acting independently, these processes interact throughout the transplant course, continuously modifying the graft microenvironment and the nature of the humoral immune response.

In light of this, the formation of non-HLA antibodies should be interpreted as the product of a continuously evolving graft immune environment, rather than as a discrete or isolated antibody response [2,7]. Initial graft injury promotes the exposure of novel antigenic targets and sustains inflammatory signaling, thereby creating conditions that favor multiple, often overlapping pathways of antibody generation [3,10]. Understanding these mechanisms is essential for interpreting whether non-HLA antibodies function primarily as pathogenic mediators of allograft injury, biomarkers of dysregulated humoral immunity, or both [9,11].

### 2.1. Exposure to Genetically Disparate Non-HLA Alloantigens

Not all non-HLA antibodies are autoantibodies. A subset develops through classical alloimmune recognition of genetically disparate donor antigens outside the HLA system. The best-characterized examples include MICA and GSTT1, although the nature of the genetic disparity differs between these two targets [12,13]. MICA is a highly polymorphic cell-surface antigen and may be recognized as foreign when donor and recipient differ; both MICA mismatch and anti-MICA antibodies have been associated with kidney transplant rejection and adverse graft outcomes [13,14].

In contrast, GSTT1 mismatch generally reflects a presence–absence polymorphism, most notably when a GSTT1-null recipient is exposed to GSTT1 expressed by the donor. The clinical relevance of this mismatch was initially demonstrated in liver transplantation, where it was identified as a risk factor for de novo immune hepatitis [15], and was subsequently considered more broadly across solid-organ and hematopoietic stem-cell transplantation [12].

Donor–recipient disparities involving non-HLA antigens may engage conventional adaptive immune responses, including antigen presentation, T-cell help, germinal-center formation, affinity maturation, and differentiation into memory B cells and antibody-secreting plasma cells [11,16]. These references provide a general immunological framework rather than direct evidence that every stage has been demonstrated specifically for MICA- and GSTT1-directed responses. Thus, antibodies against genetically disparate non-HLA antigens represent an extension of humoral alloimmunity beyond the HLA system rather than a distinct immunological phenomenon [3,7]. However, kidney-specific evidence for GSTT1 mismatch and anti-GSTT1 antibodies remains limited and inconsistent, and interpretation requires careful evaluation of donor–recipient genotype and antibody specificity [17,18,19].

From a research perspective, evaluating non-HLA alloantigens requires precise donor–recipient genotyping rather than relying solely on antibody detection panels. Investigators should focus on verifying true genetic disparities, such as genomic deletions in the case of GSTT1 null phenotypes, to robustly distinguish classical non-HLA alloimmunity from non-specific antibody binding or secondary autoimmune responses.

### 2.2. Ischemia–Reperfusion Injury and Exposure of Cryptic Antigens

Although alloantigen recognition contributes to non-HLA antibody formation, many clinically relevant non-HLA antibodies arise through fundamentally different mechanisms. Kidney transplantation inevitably exposes the allograft to ischemia–reperfusion injury (IRI), one of the earliest triggers of innate and adaptive immune activation and a major determinant of delayed graft function [20,21,22].

Beyond initiating inflammation, IRI profoundly reshapes the antigenic landscape of the transplanted organ. Cellular hypoxia, oxidative stress, complement activation, apoptosis, extracellular matrix remodeling, and the release of damage-associated molecular patterns (DAMPs) promote endothelial activation. These processes expose or release intracellular and matrix-associated molecules that are normally concealed from immune surveillance [21,22]. Once exposed, these cryptic self-antigens may become immunogenic within a highly inflammatory microenvironment [3].

Candidate targets include vimentin, collagen, fibronectin, and LG3 fragments, all of which have been implicated in non-HLA humoral immune responses following transplantation [3,7,23]. Beyond generating individual antigenic targets, IRI establishes a pro-inflammatory environment that sustains antigen presentation and favors subsequent humoral immune responses.

Extracellular vesicles may provide an additional link between graft injury and non-HLA humoral immunity. Donor kidneys release vesicles during retrieval, preservation, ischemia–reperfusion and after implantation these vesicles can carry membrane-bound and intracellular autoantigens, including injury-associated matrix components, and may facilitate antigen transfer and presentation to recipient immune cells. Experimental studies further suggest that selected apoptotic vesicle populations can promote intragraft inflammatory organization and loss of B-cell tolerance, although their contribution to human non-HLA antibody formation remains to be established [6]. Although largely experimental, these observations suggest a plausible mechanism connecting graft injury with antigen dissemination and subsequent non-HLA humoral responses. Clinically, these insights underscore that the window of initial graft injury represents a critical phase for antigen shedding. Monitoring strategies should therefore consider the baseline ischemic time and the development of delayed graft function as primary clinical indicators that may predict a higher subsequent risk of autoantibody formation against structural components like vimentin or matrix fragments.

### 2.3. Endothelial Injury and Neoantigen Generation

The vascular endothelium is the primary interface for immunological recognition and a principal target of injury during kidney transplantation. In response to ongoing inflammatory stimuli, complement activation and cellular stress, endothelial cells undergo profound phenotypic and structural alterations. These changes include the upregulation of adhesion molecules, modified surface receptor expression, and localized extracellular matrix remodeling. Consequently, the antigenic profile of the graft is altered through the exposure of previously hidden self-antigens and the generation of neoepitopes within the microenvironment [3,21].

Among the best-characterized endothelial-associated non-HLA targets are AT1R, ETAR, and LG3 fragments [7,10,23,24]. Consequently, endothelial injury not only represents a consequence of immune-mediated graft damage but also serves as a source of novel antigenic stimuli capable of sustaining humoral immune responses.

### 2.4. Loss of Self-Tolerance and Autoimmunity

Whereas antibodies directed against polymorphic donor antigens arise through classical alloimmune recognition, many non-HLA antibodies recognize self or tissue-associated antigens and therefore imply a breakdown of immunological tolerance. Under physiological conditions, central and peripheral tolerance mechanisms eliminate, edit, or functionally silence autoreactive B-cell clones. However, persistent inflammation, repeated tissue injury, endothelial activation, and exposure of normally sequestered, modified, or injury-induced tissue antigens within the graft microenvironment may progressively weaken these regulatory mechanisms [3,7,10,23].

Several immunological processes may contribute to this loss of tolerance, including bystander activation, epitope spreading, impaired immune regulation, and prolonged antigenic stimulation during chronic tissue injury. Once tolerance is disrupted, autoreactive B cells may enter germinal center reactions, undergo affinity maturation, and differentiate into memory B cells and long-lived plasma cells capable of producing high-affinity autoantibodies [7,11,25,26].

Under these conditions, alloimmunity and secondary autoimmunity may evolve in parallel after transplantation, providing a mechanistic basis for the development of non-HLA antibodies against antigens exposed, modified, or rendered immunogenic during graft injury. To better delineate this breakdown of tolerance, future mechanistic trials must transition from cross-sectional antibody testing to tracking the longitudinal evolution of autoreactive memory B-cell clones. This approach is essential to understand whether secondary autoimmunity can be arrested before transitioning into fixed, long-lived plasma cell responses.

### 2.5. Chronic Inflammation, Epitope Spreading, and Dysregulated Humoral Immunity

Persistent inflammatory signaling within the transplanted kidney provides a mechanistic link between ongoing tissue injury and the expansion of humoral immune responses. Subclinical alloimmune activity, ischemia–reperfusion-related injury, endothelial activation, infection, and extracellular matrix remodeling generate a continuous source of antigenic stimulation that may sustain antigen presentation, T-cell help, and prolonged germinal center activity [7,11,16,18,19]. Under these conditions, antibody responses may progressively broaden through epitope spreading, extending beyond initially dominant targets to include additional polymorphic, endothelial, and injury-exposed non-HLA antigens [2,25,26,27,28].

This process has important implications for the interpretation of non-HLA antibody positivity. In many recipients, non-HLA antibodies are unlikely to arise as isolated serological events but may emerge within a broader context of persistent humoral immune activation and repertoire diversification. It is hypothesized that this serological pattern may be associated with an expansion of antigen-experienced and memory B-cell compartments, sustained plasma-cell responses, a potential reduction in naïve B-cell pools, and impaired regulatory B-cell activity. These relationships remain to be formally demonstrated in prospective longitudinal cohorts, as direct evidence linking these cellular phenotypes to the development and evolution of non-HLA antibody repertoires remains limited [29,30,31,32].

In the future, validating this underlying mechanism will require robust data from longitudinal B-cell phenotyping studies, high-throughput antibody repertoire sequencing and specific markers of intragraft plasma-cell persistence.

Accordingly, the accumulation of multiple non-HLA antibody specificities may indicate a dysregulated humoral immune state, but should not be regarded as direct evidence of a defined B-cell phenotype.

## 3. Evaluating the Pathogenic Potential of Non-HLA Antibodies

The pathogenic significance of non-HLA antibodies remains one of the most debated questions in transplantation immunology [9,33]. Although numerous studies have reported associations between non-HLA antibody positivity and adverse transplant outcomes, association alone does not establish causality [3,28]. Demonstrating pathogenicity requires converging mechanistic, experimental, clinicopathological, and clinical evidence rather than simple associations with adverse graft outcomes. However, the strength of this evidence varies considerably among individual antibody specificities [7,33]. Whereas some antibodies are supported by robust mechanistic and clinical evidence, others have been described primarily through observational studies and remain incompletely characterized. Therefore, non-HLA antibodies should not be regarded as a homogeneous group with equivalent pathogenic potential; rather, each antibody specificity should be evaluated according to the strength, consistency, and biological plausibility of the available evidence. In this narrative review, terms such as strong, intermediate, limited, and emerging evidence are used descriptively to indicate the degree of convergence among mechanistic, experimental, clinicopathological, and clinical data, rather than as part of a formal evidence-grading system. An overview of the antibodies discussed in this section, together with their principal biological effects and current clinical interpretation, is provided in Table 1.

### 3.1. Functional Antibodies and Receptor-Mediated Cellular Activation

Among non-HLA antibodies, those directed against the angiotensin II type 1 receptor (AT1R) provide the most compelling evidence for direct pathogenicity. Unlike many conventional alloantibodies, which commonly mediate injury through complement activation or Fc receptor-dependent effector mechanisms, AT1R antibodies can function as agonistic autoantibodies that directly activate their target receptor. Experimental studies have demonstrated that AT1R antibody-mediated receptor activation initiates intracellular signaling pathways analogous to those triggered by angiotensin II, promoting endothelial activation, tissue factor expression, pro-inflammatory cytokine production, vasoconstriction, cellular proliferation, and vascular remodeling [3,24,34].

The landmark study by Dragun and colleagues linked AT1R-activating antibodies to severe steroid-resistant vascular rejection in kidney transplant recipients without detectable HLA-DSAs and demonstrated receptor-mediated vascular signaling, providing compelling clinical and mechanistic evidence of direct pathogenicity [24]. Subsequent studies have associated AT1R antibody positivity with rejection, microvascular inflammation, impaired graft function, and inferior graft survival in adult and pediatric kidney transplant recipients [9,35,36,37]. The convergence of mechanistic, experimental, histopathological, and clinical evidence therefore makes AT1R antibodies the best-supported example of a directly pathogenic non-HLA antibody.

Antibodies directed against the endothelin-1 type A receptor (ETAR) represent another group of potentially functional receptor-targeting antibodies. ETAR is a G protein-coupled receptor involved in the regulation of vascular tone, endothelial homeostasis, leukocyte recruitment, and vascular remodeling. Experimental evidence indicates that antibodies targeting AT1R and ETAR can activate endothelial signaling through β2-arrestin- and mTOR-associated pathways, providing a biologically plausible mechanism of antibody-mediated endothelial injury [34].

Clinically, ETAR antibodies have been associated with vascular inflammation and declining graft function, particularly when detected together with AT1R antibodies in pediatric kidney transplant recipients. In the cohort reported by Pearl et al., concurrent AT1R and ETAR antibody positivity was associated with a greater than 30% decline in eGFR, arteritis, and elevated IL-8 levels, but not with rejection, development of de novo HLA-DSAs, or allograft loss [38]. These findings support a relationship with vascular inflammation and functional decline but do not establish an independent association with rejection or graft failure. The available evidence for ETAR antibodies remains substantially less extensive and less standardized than that for AT1R antibodies. ETAR antibodies should therefore be considered biologically plausible and potentially pathogenic, but still investigational in kidney transplantation.

### 3.2. Endothelial Activation and Microvascular Injury

Several non-HLA antibodies recognize antigens expressed on endothelial cells or molecules exposed following endothelial injury. More broadly, antibodies directed against endothelial antigens have been associated with allograft rejection in kidney transplantation [39]. Accumulating clinical evidence has also demonstrated associations between selected non-HLA antibodies and histological features of antibody-mediated rejection, including glomerulitis, peritubular capillaritis, transplant glomerulopathy, and intimal arteritis [3,7,9,27,40]. Some of these associations persist after adjustment for HLA-DSA status, suggesting that selected non-HLA antibodies may contribute to endothelial injury independently of conventional HLA-directed alloimmunity [9,40].

Beyond these clinicopathological observations, experimental studies indicate that several non-HLA antibodies can directly activate endothelial cells, promoting leukocyte adhesion, cytokine production, procoagulant signaling, and vascular inflammation [3,10,34]. Although the strength of evidence varies among individual antibody specificities, these findings provide a biologically plausible link between circulating non-HLA antibodies and the endothelial dysfunction underlying chronic allograft injury. Interpretation of non-HLA antibodies must remain contextual, as their pathogenic potential varies substantially across clinical settings, assay platforms, and coexisting alloimmune activity.

### 3.3. Antibodies Against Genetically Disparate Non-HLA Alloantigens

Not all pathogenic non-HLA antibodies are directed against self-antigens. Additional evidence supporting antibody-mediated pathogenicity comes from antibodies recognizing genetically disparate donor-derived antigens, which arise through classical alloimmune mechanisms analogous to those responsible for HLA-DSA formation. The best-characterized examples are MICA and GSTT1, although the nature of the donor–recipient disparity differs between them.

In a landmark multicentre study, Zou and colleagues reported that anti-MICA antibodies were associated with rejection even among recipients who were well matched at HLA loci [13]. More recently, Carapito and colleagues provided genetic and serological evidence supporting MICA as a clinically relevant histocompatibility antigen in kidney transplantation [14].

Subsequent studies have associated anti-MICA antibodies with acute rejection, chronic allograft injury, transplant glomerulopathy, and inferior graft outcomes, although the magnitude and reproducibility of these associations have varied across studies [3,7,41].

Similarly, donor–recipient GSTT1 mismatch may induce alloantibody formation through conventional adaptive immune mechanisms. Although the clinical relevance of this mismatch was first characterized in liver transplantation [12,15], kidney-specific studies have shown that GSTT1-null recipients of GSTT1-positive kidneys can develop anti-GSTT1 antibodies [17], and donor-specific anti-GSTT1 antibodies have been detected in selected recipients with rejection and C4d deposition [18]. However, anti-GSTT1 antibodies have not consistently been associated with impaired graft function or graft loss, and the available studies remain small and heterogeneous [17,18,19,42].

Rare alloantibodies against human neutrophil antigen-3a (HNA-3a) have also been reported in kidney transplant recipients with otherwise unexplained positive T- and B-cell flow crossmatches and no detectable HLA-DSA. Evidence is limited to small case series, and the rarity of susceptible HNA-3b homozygous recipients restricts the broader clinical utility of HNA-3a testing [19].

Collectively, these observations demonstrate that humoral alloimmunity is not restricted to HLA antigens but also encompasses genetically disparate non-HLA targets capable of eliciting clinically relevant antibody responses. However, unlike receptor-targeting antibodies such as AT1R, evidence for MICA- and especially GSTT1-specific antibodies rests largely on clinical association studies, whereas the biological processes linking antibody recognition to allograft injury remain incompletely understood.

### 3.4. Autoantibodies Generated Following Tissue Injury

A distinct group of non-HLA antibodies recognizes self-antigens that become exposed or are released following ischemia–reperfusion injury, apoptosis, extracellular matrix remodeling, or chronic inflammation. Unlike antibodies directed against polymorphic donor antigens, these autoantibodies arise secondary to tissue injury and the loss of self-tolerance, thereby linking graft injury with subsequent humoral immune responses.

Among the best-characterized examples are antibodies directed against perlecan-derived LG3 fragments. Experimental and clinical studies have shown that vascular injury promotes the release of LG3, whereas pre-existing or circulating anti-LG3 antibodies may amplify vascular inflammation, increase susceptibility to ischemia–reperfusion injury, and predispose to acute and chronic allograft rejection [23,43]. Nevertheless, clinical findings have not been uniform, and some studies have not demonstrated an association between humoral responses to perlecan/LG3 and chronic immune-mediated injury [19].

Experimental and clinical observations implicate anti-vimentin antibodies in allograft injury, but much of the mechanistic evidence derives from non-renal models, and kidney-specific studies have reported conflicting associations with chronic allograft injury and interstitial fibrosis and tubular atrophy [19,44,45,46]. Differences in sampling time, patient selection, and assay methodology may contribute to these inconsistencies. Anti-vimentin antibodies may therefore reflect or amplify ongoing tissue injury, but their independent pathogenic significance in human kidney transplantation remains uncertain [7,19].

Other endothelial and extracellular matrix antibodies, including antibodies against fibronectin, collagen, agrin, and related antigens, remain best regarded as emerging because the available evidence is heterogeneous [3,19,27]. These targets are often assessed within broad antibody panels, using different assay platforms and positivity thresholds, and in relatively small or clinically diverse cohorts. In contrast to receptor-targeting antibodies such as AT1R, direct target-specific signaling mechanisms have not been consistently defined, and it remains difficult to separate causal pathogenicity from secondary antigen exposure during tissue injury. Their current value may therefore lie in identifying patterns of ongoing endothelial or matrix remodeling rather than supporting routine single-antibody clinical decisions.

Natural antibodies represent a related but biologically distinct form of non-HLA reactivity. These polyreactive IgM and IgG antibodies recognize apoptotic cells and oxidation-associated epitopes and are produced in part by innate-like B-cell populations. Cohort studies summarized by Ryeed et al. linked selected natural-antibody profiles with microvascular injury, rejection phenotypes, or graft loss, but associations were not uniform across studies and the underlying mechanisms remain uncertain. Their presence may therefore indicate tissue stress or heightened immune activation as well as possible effector activity [6].

Collectively, these findings support the concept of a self-perpetuating cycle in which graft injury promotes autoantibody formation, while autoantibody-mediated immune responses sustain inflammation and vascular remodeling.

**Table 1:** Major Non-HLA Antibodies in Kidney Transplantation: Biological Characteristics and Current Clinical Interpretation.

Antibody and Target	Biological Category	Principal Evidence	Current Clinical Interpretation	References
AT1R: angiotensin II type 1 receptor	Functional receptor-targeting autoantibody	Receptor activation, endothelial inflammation, vasoconstriction and remodeling; associated with vascular rejection, MVI, dysfunction, and graft loss.	Strongest converging evidence for direct pathogenicity. Testing may complement assessment of selected HLA-DSA-negative AMR or unexplained vascular injury. Evaluated primarily via commercial solid-phase ELISA; moderate standardization exists, but validated clinical thresholds vary.	[9,24,34,35,36,37]
ETAR: endothelin-1 type A receptor	Functional receptor-targeting autoantibody	Endothelial dysfunction and vascular inflammation; associated with arteritis and declining graft function, often with AT1R antibodies.	Biologically plausible, but supported by less extensive evidence than AT1R antibodies. Testing relies mainly on solid-phase ELISA, with limited assay standardization and no broadly validated clinical cutoff. ETAR antibodies therefore remain under investigation.	[34,38]
MICA: major histocompatibility complex class I- related chain A	Polymorphic non-HLA alloantigen- directed antibody	Donor-specific alloimmune response; associated with rejection, transplant glomerulopathy, chronic injury and graft loss.	Extends alloimmunity beyond HLA. Interpretation requires donor specificity and an appropriate clinical context. Detected using multiplex bead-based assays (Luminex); standardization is limited by variable antigen presentation on beads.	[13,14,41]
LG3: perlecan derived laminin G-like fragment 3	Injury-associated autoantibody	May amplify ischemia–reperfusion injury and vascular inflammation; associated with acute rejection and chronic vascular injury.	May link tissue injury with humoral activation; independent clinical and prognostic value requires validation. Measured via research-grade ELISA or multiplex beads; currently unstandardized for routine clinical use.	[23,43]
GSTT1: glutathione S-transferase theta-1	Mismatch-associated non-HLA alloantigen-directed antibody	Alloimmune response from donor–recipient genetic mismatch; kidney-specific evidence remains limited and inconsistent.	Requires confirmation of mismatch and antibody specificity. Its role in kidney transplantation remains investigational. Requires donor–recipient genotyping paired with enzyme-linked binding assays; unstandardized in renal cohorts.	[12,15,17,18,19,42]
Vimentin: vimentin	Injury-associated autoantibody	Secondary response to tissue injury and loss of self-tolerance; associated with chronic rejection, vasculopathy, and inferior outcomes.	May contribute to injury or reflect tissue damage and broader humoral activation. Independent pathogenicity remains uncertain. Assessed through non-commercial solid-phase assays; poor inter-laboratory reproducibility.	[23,44,45,46]
Other endothelial and extracellular matrix antibodies: fibronectin, collagen, agrin, and related targets	Predominantly injury-associated autoantibodies	Heterogeneous responses associated with endothelial injury, matrix remodeling, MVI and chronic allograft injury.	Insufficient evidence for routine single-antibody testing or treatment decisions. Tested via highly heterogeneous, non-standardized research platforms; lack reproducible reference intervals.	[7,19,27,43,44,45,46]

Abbreviations: AMR, antibody-mediated rejection; AT1R, angiotensin II type 1 receptor; ETAR, endothelin-1 type A receptor; GSTT1, glutathione S-transferase theta-1; HLA-DSA, donor-specific antibody against human leukocyte antigens; LG3, perlecan-derived laminin G-like fragment 3; MICA, major histocompatibility complex class I-related chain A; MVI, microvascular inflammation.

### 3.5. Interaction Between Non-HLA Antibodies and HLA Donor-Specific Antibodies

The biological significance of non-HLA antibodies cannot be fully understood without considering their relationship with HLA-DSAs. Non-HLA antibodies may be detected in isolation, coexist with HLA-DSAs, or occur in recipients with microvascular inflammation despite the absence of detectable circulating HLA-DSAs. These settings are biologically and clinically distinct and should not be interpreted as representing an equivalent form of humoral allograft injury [3,7,9].

#### 3.5.1. Isolated Non-HLA Antibody Positivity

The detection of non-HLA antibodies in the absence of HLA-DSAs does not, by itself, establish an active pathogenic process. Some non-HLA antibodies may be present before transplantation as a consequence of previous tissue injury, inflammation, infection, autoimmunity, or naturally occurring autoreactivity. Others may persist after transplantation through memory B-cell activation and long-lived plasma-cell responses, even in the absence of ongoing graft injury [3,30,31].

Nevertheless, selected non-HLA antibodies may be clinically relevant in the absence of detectable HLA-DSAs. AT1R antibodies provide the best-supported example, particularly when their detection is accompanied by vascular, histological, or molecular evidence compatible with receptor-mediated injury [9,24,35,36].

Therefore, isolated non-HLA antibody positivity may be clinically relevant when the detected antibody has a biologically plausible effector mechanism and is accompanied by compatible vascular, histological, or molecular evidence of injury. In the absence of such findings, however, isolated positivity should be interpreted cautiously because assay thresholds, antibody levels, timing of detection, and clinical associations remain insufficiently standardized [9,28,33].

#### 3.5.2. Coexistence of Non-HLA Antibodies and HLA-DSAs

Non-HLA antibodies frequently coexist with HLA-DSAs, making it difficult to determine the contribution of each antibody system to graft injury. In this setting, non-HLA antibodies may not necessarily represent independent causes of rejection but may act as modifiers or amplifiers of an established HLA-directed alloimmune response [3,9,10].

Several biological mechanisms could explain such interaction. HLA-DSA binding may promote endothelial activation, complement-dependent or Fc receptor-dependent inflammation, and microvascular injury. The resulting tissue damage may expose cryptic self-antigens, modify extracellular matrix components, and generate neoepitopes that subsequently stimulate non-HLA antibody responses. Conversely, receptor-targeting non-HLA antibodies may promote endothelial activation, vasoconstriction, inflammatory signaling, and vascular remodeling, potentially increasing endothelial susceptibility to injury mediated by HLA-DSAs [10,23,34].

Clinical studies have reported more severe rejection phenotypes or poorer graft outcomes in some recipients in whom AT1R antibodies coexist with HLA-DSAs compared with recipients with only one antibody type [9,36,40]. Similarly, ETAR antibodies commonly occur together with AT1R antibodies and may be associated with vascular inflammation and declining graft function, suggesting that multiple receptor-directed and HLA-directed antibody responses may converge at the level of endothelial injury [34,38].

However, these observations should not automatically be interpreted as proof of biological synergy. Demonstration of synergy requires evidence that the combined effect of HLA-DSAs and non-HLA antibodies exceeds the sum of their individual effects. Most available studies are observational, include relatively small or selected populations, and do not formally test statistical interaction. In addition, differences in HLA-DSA strength, class, complement-binding capacity, antibody timing, histological severity, and treatment may confound the reported associations [9,28,33].

Accordingly, the coexistence of HLA-DSAs and non-HLA antibodies is best described as having potentially additive, cooperative, or mutually amplifying effects, whereas true biological synergy remains to be established.

#### 3.5.3. Non-HLA Antibodies in HLA-DSA-Negative Microvascular Inflammation

A particularly important clinical setting is the presence of microvascular inflammation in recipients without detectable circulating HLA-DSAs. Non-HLA antibody reactivity has also been detected in HLA-DSA-negative recipients whose biopsies show microvascular or vascular lesions compatible with antibody-mediated injury [2,27,40].

In this context, receptor-targeting antibodies such as AT1R and ETAR provide a plausible mechanism of endothelial injury because they can activate intracellular vascular signaling independently of HLA recognition and, in the case of AT1R antibodies, often without prominent complement deposition [9,24,34]. Antibodies against MICA or injury-exposed endothelial and extracellular matrix antigens may also contribute to, or reflect, microvascular injury, although the evidence supporting their independent pathogenicity is less consistent [7,13,23,27].

Broad non-HLA antibody repertoires may be particularly informative in this setting. The pretransplant burden and diversity of non-HLA antibodies have been associated with the subsequent development of histological features of antibody-mediated rejection, supporting the possibility that repertoire-level humoral activity may identify recipients at increased risk even when individual antibody specificities are not clearly pathogenic [2]. Nevertheless, HLA-DSA-negative microvascular inflammation should not automatically be attributed to non-HLA antibodies. Detectable circulating HLA-DSAs may be absent because of assay sensitivity, low antibody concentration, temporal variation, or other technical and biological factors. Moreover, microvascular inflammation may result from antibody-independent mechanisms, including natural killer cell activation, innate immune responses, infection, ischemic injury, thrombotic microangiopathy, recurrent disease, or T-cell-mediated endothelial injury [6,33].

Non-HLA antibodies should therefore be considered one possible contributor to HLA-DSA-negative microvascular inflammation rather than a universal explanation for this heterogeneous phenotype.

#### 3.5.4. Interpretation in Relation to HLA-DSA Status

Interpretation of non-HLA antibodies should therefore be anchored to HLA-DSA status and compatible clinicopathological evidence. In HLA-DSA-negative recipients, selected non-HLA antibodies may support a humoral or endothelial process but cannot independently establish antibody-mediated rejection. In HLA-DSA-positive recipients, they may indicate a broader humoral response and potentially amplify the severity or persistence of endothelial injury [2,6,28].

### 3.6. Reported Frequency and Distribution of Non-HLA Antibodies

The population-level prevalence of non-HLA antibodies in kidney transplantation remains poorly defined. Reported detection rates differ considerably according to the antibody specificity, study population, sampling time, assay platform, and threshold used to define positivity. For AT1R antibodies, adult pretransplant studies have reported rates ranging from approximately 8% to 47%, with much of this variability attributable to the use of different thresholds [9,35,36]. In a longitudinal cohort of 65 pediatric kidney transplant recipients, AT1R antibodies were detected in 58% at one or more time points from before transplantation to two years after transplantation; 23% had pretransplant antibodies and 26% developed them de novo [37]. In the same pediatric population, concurrent AT1R and ETAR antibody positivity was reported in approximately 35% [38]. These findings suggest that receptor-targeting antibodies may be detected more frequently in pediatric recipients, although direct adult–pediatric comparisons remain limited.

For MICA antibodies, a large multicentre study identified pretransplant antibodies in 217 of 1910 kidney transplant recipients, corresponding to 11.4% [13]. Reliable prevalence estimates are not available for LG3, GSTT1, vimentin, or most other endothelial and extracellular matrix antibodies. Evidence for these specificities is derived mainly from relatively small or selected cohorts enriched for rejection, microvascular inflammation, sensitization, or graft dysfunction [2,17,18,19,23,40,41,42,43,44,45,46]. GSTT1 antibody formation additionally depends on an appropriate donor–recipient genetic mismatch and therefore cannot be described by a single prevalence estimate applicable to all recipients. Large registries do not routinely collect non-HLA antibody data, and robust comparisons among stable recipients, patients with HLA-DSA-negative AMR, and unselected transplant populations are lacking. Published percentages should therefore be interpreted as study-specific detection rates rather than definitive population prevalence estimates. The principal assays, methodological limitations, and clinical interpretations are summarized in Table 1.

### 3.7. Assessing the Pathogenicity of Non-HLA Antibodies

The evidence reviewed above supports a hierarchy rather than a uniform assumption of pathogenicity. AT1R antibodies have the strongest converging mechanistic, experimental, histopathological, and clinical support [9,24,34,35,36]. Evidence for ETAR antibodies is biologically plausible but less extensive and standardized [34]. MICA-specific antibodies have intermediate but heterogeneous clinical support [13,14,41], whereas evidence for GSTT1 antibodies in kidney transplantation remains limited and inconsistent [17,18,19,42]. The pathogenic significance of LG3, vimentin, collagen, fibronectin, agrin, and related injury-associated antibodies remains heterogeneous and context-dependent [7,19,23,43,44,45,46].

Several limitations temper these conclusions. Most studies are observational or retrospective and often enrich for graft dysfunction, microvascular inflammation, or histological injury, potentially inflating positive associations; findings from pediatric or otherwise selected cohorts may also have limited generalizability to adult kidney transplant recipients [28,33,38,39,40]. Analytical heterogeneity is equally important. AT1R/ETAR ELISAs and multiplex bead-based or protein-array platforms differ in antigen presentation, normalization, calibration, background reactivity, and positivity criteria. Recombinant or immobilized antigens may not preserve native conformation or relevant post-translational modifications and may alter epitope accessibility, especially for multispanning receptors. Consistent with these concerns, commercial multiplex platforms show poor concordance even for shared targets and should not be considered interchangeable [47]. This platform dependence was also evident in a recent multicenter pilot project in which two commercial multiplex assays identified different pre- and post-transplant candidate antibody signatures, although persistence of non-HLA reactivity across time was associated with rejection [19]. Cell-based endothelial crossmatch assays complement solid-phase methods by detecting antibodies against native or unidentified cell-surface targets. Their relevance depends on the cellular substrate: endothelial progenitor cells, HUVECs, and immortalized lines may not reproduce the antigenic profile of renal microvascular endothelium, while HLA expression can confound attribution of reactivity to non-HLA antibodies. Donor-derived or kidney-specific HLA-deficient platforms may improve tissue relevance and assessment of donor-reactive antibodies, but remain technically demanding and insufficiently standardized [48]. Interpretation also depends on thresholds, timing, and donor specificity. No universal cutoffs have been validated, and low-level reactivity against some non-HLA targets may occur in individuals without clinically apparent graft injury. Indeed, manufacturer-defined thresholds in some bead-based platforms have yielded extensive positivity in healthy control cohorts, and reactivity may vary with demographic and sensitization characteristics. These findings emphasize the need for population-appropriate reference distributions and outcome-validated, target-specific thresholds [2,6,28,49]. Pretransplant antibodies may reflect prior sensitization, autoreactivity, or tissue injury, whereas de novo antibodies may accompany evolving graft injury, and single-time-point measurements cannot establish directionality. Donor specificity can be assessed for genetically disparate targets such as MICA and GSTT1 only when donor antigen or mismatch data are available, whereas AT1R, ETAR, LG3, and vimentin generally recognize non-polymorphic self-antigens. Limited inter-laboratory reproducibility and the absence of outcome-validated thresholds currently preclude uniform interpretation across centers [2,6,48,49].

An additional target-specific limitation concerns MICA. Routine lymphocyte-based crossmatches may fail to detect clinically relevant anti-MICA reactivity because MICA is not constitutively expressed on the lymphocytes typically used in conventional crossmatching. Moreover, incomplete representation of MICA specificities on commercial panels and the absence of donor and recipient MICA genotyping in many studies may prevent confirmation that detected antibodies are donor-specific [19,48].

These design and analytical limitations make association insufficient evidence of independent pathogenicity. Causality requires temporal precedence, reproducible associations after adjustment for HLA-DSAs and other confounders, target-specific biological activity, a compatible clinicopathological phenotype, and ideally experimental or interventional evidence that altering the antibody response changes graft injury or outcome [3,6,33].

Accordingly, selected antibodies, most notably AT1R, may function as pathogenic effectors, whereas broader or injury-associated patterns may primarily reflect humoral immune activation [2,6,7,24,28]. This dual interpretation is developed in Section 4, and Table 1 summarizes the major antibodies and their current clinical interpretation.

### 3.8. Evidence from Experimental Animal Models

Experimental animal models provide direct evidence that selected non-HLA antibodies can contribute to vascular and allograft injury under defined conditions. In a rat renal-transplant model, passive infusion of human AT1R antibodies induced vascular changes and increased blood pressure, supporting a receptor-mediated pathogenic effect [24]. In a murine model of renal ischemia–reperfusion injury, anti-LG3 antibodies aggravated renal dysfunction and microvascular injury, providing evidence that tissue injury may create a permissive environment in which these antibodies amplify graft damage [43]. In a murine cardiac-allograft model, preimmunization with vimentin accelerated rejection and increased inflammatory and microvascular injury. This effect was absent in B-cell-deficient recipients and was restored by transfer of anti-vimentin-containing serum, supporting an antibody-dependent contribution in the presence of an alloimmune response [44].

Collectively, these models support the pathogenic potential of selected non-HLA antibodies but do not establish that every antibody detected in human recipients is independently pathogenic. Experimental antibody induction or passive transfer may not reproduce the concentration, timing, or immune context observed clinically, and findings from non-renal transplant models require cautious extrapolation to kidney transplantation.

These experimental findings strengthen the evidence for selected antibody specificities while reinforcing the need to distinguish direct pathogenicity from antibody responses that may primarily reflect tissue injury or broader humoral activation.

## 4. Beyond Pathogenicity: Interpreting Non-HLA Antibodies in the Context of Humoral Immunity

Having considered target-specific pathogenicity and the limits of causal inference, this section asks whether the overall pattern of non-HLA reactivity provides information beyond the effects of individual antibodies.

### 4.1. From Individual Antibodies to Repertoire-Level Humoral Signals

Non-HLA antibodies frequently occur as multiple simultaneous specificities rather than isolated responses. High-dimensional studies suggest that the overall burden and persistence of non-HLA antibody reactivity may be more informative than individual specificities in some cohorts. However, this finding has not been consistently replicated and appears dependent on the assay platform, study population, sampling time, and analytical thresholds [2,8,19,28].

Recent longitudinal evidence reinforces the potential importance of antibody trajectories but does not establish a uniform prognostic pattern. In one study summarized by Ryeed et al., global non-HLA reactivity was not associated with AMR at either the pre- or post-transplant time point, yet overall reactivity declined after transplantation only among recipients with functioning grafts. This observation supports serial assessment as a means of capturing changes in immune activation, while requiring confirmation in larger prospective cohorts [6].

It is hypothesized that repertoire architecture may integrate the cumulative effects of antigen exposure, germinal-center selection, potential memory B-cell expansion, plasma-cell activity, and persistent humoral stimulation. This repertoire-level view is consistent with the broader principle that adaptive immune information can reside in the organization and diversity of receptor or antibody repertoires [32]. Its application to kidney transplantation remains hypothesis-generating and should not yet be treated as a validated clinical algorithm.

### 4.2. Implications for Precision Immune Monitoring

These observations support the use of non-HLA antibody profiles as complementary rather than stand-alone biomarkers of graft injury and humoral activity. An integrated assessment may combine non-HLA antibody profiles with HLA donor-specific antibodies, histopathology, molecular diagnostics, donor-derived cell-free DNA, graft function, and immune phenotyping. Histology localizes and classifies tissue injury, molecular platforms identify rejection-associated transcriptional states, and donor-derived cell-free DNA provides a dynamic signal of graft injury [2,50,51]. Non-HLA antibody profiles may add mechanistic context by indicating receptor-targeting, broad humoral, or predominantly injury-induced responses [2]. Such integration could improve risk stratification and inform biopsy timing, surveillance intensity, and clinical decision-making.

### 4.3. Clinical Utility of Non-HLA Testing

To translate these heterogeneous data into clinical practice, current non-HLA evaluations can be organized into a practical classification based on their current clinical applicability:

(a) Complementary Clinical Indicators (e.g., AT1R):

Testing is not indicated for universal screening but can provide useful complementary information in highly selective scenarios. These include the diagnostic evaluation of biopsy-proven HLA-DSA-negative AMR, unexplained microvascular inflammation (glomerulitis or capillaritis), or severe, steroid-refractory vascular rejection. Positive results in these specific contexts should raise suspicion of receptor-mediated endothelial activation and prompt closer post-transplant surveillance.

(b) Investigational Targets (e.g., ETAR and MICA):

These targets have shown associations with adverse graft outcomes in observational cohorts but lack validated outcome-based thresholds, assay harmonization, or uniform inter-laboratory reproducibility. Their detection may indicate a higher individual risk profile but should not independently guide immunosuppressive modifications or targeted anti-humoral therapy.

(c) Research Biomarkers (e.g., LG3, vimentin and broad panels):

These antibodies may primarily reflect cellular stress responses, exposure of cryptic structural antigens, or broader humoral activation following tissue damage. While valuable for tracking immune activation trajectories and epitope spreading in scientific trials, they currently lack the translational granularity required for routine clinical decision-making and should be regarded strictly as research tools.

## 5. Conclusions

Non-HLA antibodies should not be interpreted as a single pathogenic category. The strongest evidence supports direct effector functions for selected receptor-targeting antibodies, particularly AT1R, whereas many injury-associated specificities and broader repertoires may primarily reflect tissue damage or dysregulated humoral immunity.

We propose that selected non-HLA antibodies may contribute to a feed-forward cycle involving graft injury, secondary antigen exposure, epitope spreading, and antibody-repertoire diversification. Although biologically plausible, this model remains hypothesis-generating because direct longitudinal evidence linking non-HLA antibodies to defined changes in B-cell homeostasis is currently lacking.

The clinical value of non-HLA antibody testing will depend on assay harmonization, longitudinal validation, and demonstration that non-HLA profiles add meaningful information beyond established immunological, histological, and functional parameters.

At present, non-HLA antibody positivity alone should neither establish a diagnosis of AMR nor independently guide treatment. Routine screening with broad non-HLA antibody panels is not supported by the available evidence. Targeted testing may be considered in selected patients, particularly those with HLA-DSA-negative AMR or otherwise unexplained microvascular inflammation, provided that the findings are interpreted together with graft histology, HLA-DSA status, graft function, and the broader clinical context.

## Data Availability

No new data were created or analyzed in this study. Data sharing is not applicable to this article.
